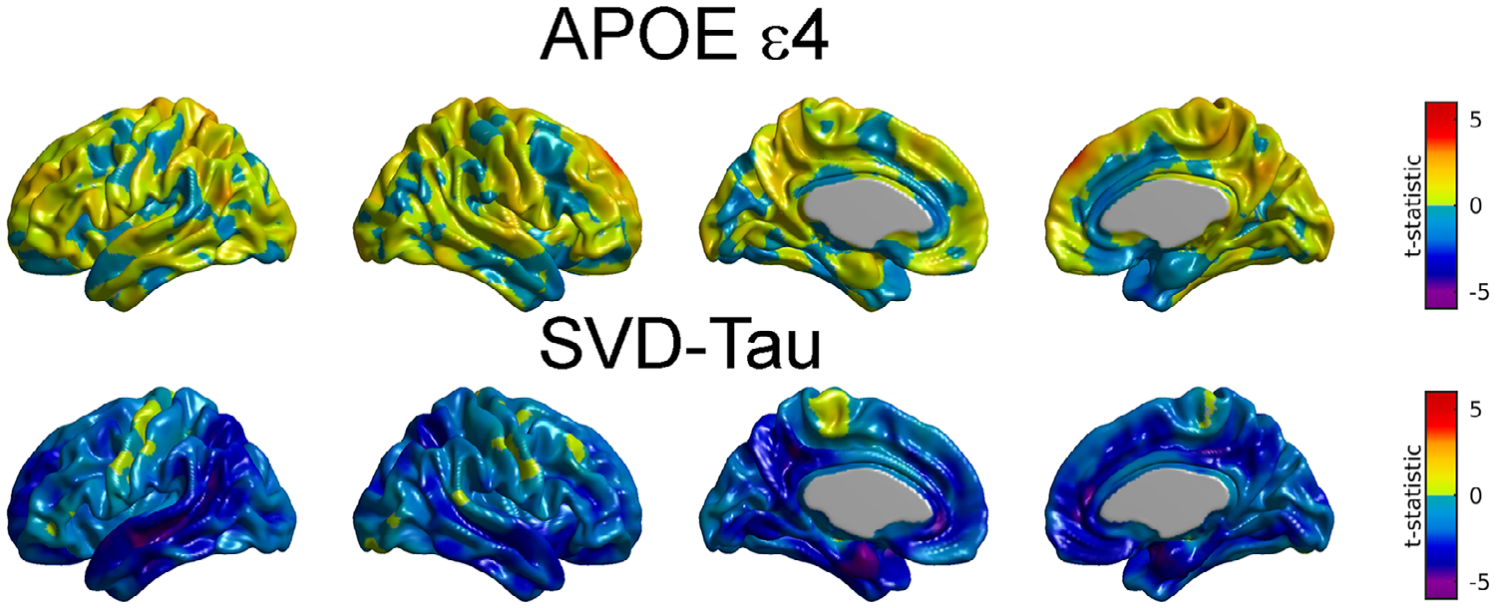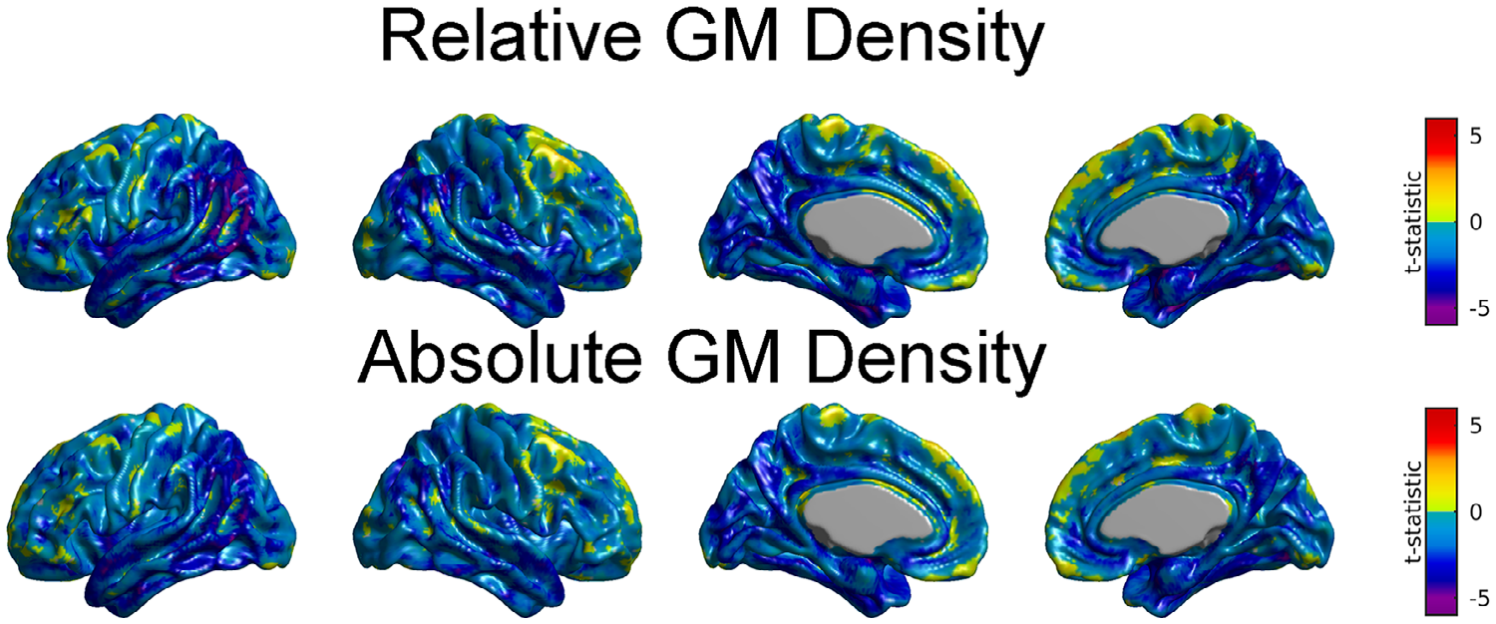# Tau is associated with atrophy in MCI independently of beta‐amyloid and APOE e4 genotype

**DOI:** 10.1002/alz70856_102268

**Published:** 2025-12-25

**Authors:** Felix Carbonell, Carolann McNicoll, Alex P Zijdenbos, Barry J Bedell

**Affiliations:** ^1^ Biospective Inc., Montreal, QC, Canada; ^2^ Biospective Inc, Montreal, QC, Canada

## Abstract

**Background:**

It has been recently shown that spatially distributed scores from Tau PET images are significantly correlated with cortical thickness and glucose metabolism in MCI [1, 2]. We hypothesize that Tau PET scores also have a similar association with other atrophy biomarkers, such as gray matter (GM) density, independently of the effects of beta‐amyloid and APOE e4 genotype.

**Method:**

Our cross‐sectional statistical analysis was applied to concurrent (within a 90‐days window) Tau PET, Amyloid PET, and anatomical MRI images from *N* = 222 MCI subjects from the ADNI study. From the MRI images, we derived different atrophy biomarkers, including cortical thickness, relative GM density (i.e. proportion of gray matter to other tissue types), and absolute GM density (i.e. modulated by the Jacobian determinants of the deformation field). We employed a Singular Value Decomposition (SVD) approach to each pair of cross‐correlation matrices between PET images and MRI‐derived maps. The resulting SVD‐based individual scores were used to fit vertexwise models for assessing the effect of tau and APOE ε4 on cortical thickness and GM density, accounting for the effect of beta‐amyloid.

**Result:**

The first SVD component accounted for 21.56%, 12.93%, 11.91% of the total co‐variability between the pairs (cortical thickness, tau), (relative GM density, tau) and (absolute GM density, tau), respectively. Figures 1A, and 1B show the t‐statistic maps for the effects of APOE e4 and SVD‐based tau scores on cortical thickness after accounting for the beta‐amyloid effects. Similarly, Figure 2A and 2B show the t‐maps for the effects of the tau scores on relative and absolute GM density, respectively.

**Conclusion:**

We determined that the distributed tau scores showed extended areas of statistical significance with cortical thickness. Although less spatially extended, the relative and absolute GM densities also showed areas of tau‐related reductions. Our results suggest that in MCI, tau is associated with consistent atrophy patterns derived from different imaging biomarkers independently of beta‐amyloid burden, reflecting a significant role of tau in neurodegeneration.

**References**

1. Carbonell *et al.*, *J. Alzheimer Dis.*,**73**: 543–557, 2020.

2. Carbonell *et al.*, *Alzheimer and Dementia*, 2025, in press.